# Preliminary Analysis of Hydrodynamic Drag Reduction and Fouling Resistance of Surfaces Inspired by the Mollusk Shell, *Dosinia juvenilis*

**DOI:** 10.3390/biomimetics9060363

**Published:** 2024-06-15

**Authors:** Benjamin W. Hamilton, O. Remus Tutunea-Fatan, Evgueni V. Bordatchev

**Affiliations:** 1 Department of Mechanical and Materials Engineering, Western University, London, ON N6A 6B9, Canada; 2 Automotive and Surface Transportation, National Research Council of Canada, London, ON N6G 4X8, Canada

**Keywords:** bio-inspired, drag reduction, fouling resistance, functional surface, mollusk shell, computational fluid dynamics, riblets

## Abstract

Many species of plants and animals show an ability to resist fouling with surface topographies tailored to their environments. The mollusk species *Dosinia juvenilis* has demonstrated the ability to resist the accumulation of fouling on its outer surface. Understanding the functional mechanism employed by nature represents a significant opportunity for the persistent challenges of many industrial and consumer applications. Using a biomimetic approach, this study investigates the underlying hydrodynamic mechanisms of fouling resistance through Large Eddy simulations of a turbulent boundary layer above a novel ribletted surface topography bio-inspired by the *Dosinia juvenilis*. The results indicate a maximum drag reduction of 6.8% relative to a flat surface. The flow statistics near the surface are analogous to those observed for other ribletted surfaces in that the appropriately sized riblets effectively reduce the spanwise and wall-normal velocity fluctuations near the surface. This study supports the understanding that nature employs ribletted surfaces toward multiple functionalities including the considered drag reduction and fouling resistance.

## 1. Introduction

Biomimetics is defined as the study of plants and animals as models for the development of advanced solutions to science and engineering challenges inspired by parallel solutions observed in nature [1]. Biomimetics is a multi-disciplinary field of research involving biologists, chemists, physicists, material scientists, and engineers. Otto Schmitt introduced the concept of ‘biomimetics’ in the late 1950s, and since its inception, this approach has led to numerous scientific breakthroughs. For instance, several drag-reducing mechanisms have been identified in nature that serve to help animals conserve energy and/or increase velocity [2]. While the true mechanisms are still not fully understood, the compliant skin of dolphins seemingly defies hydrodynamic theory by allowing a laminar boundary layer to persist along the entire length of its body [3]. By remaining in the laminar region, the propulsive power required for the dolphin to swim at high speeds is reduced. Similarly, fish-scales have been observed to delay the transition to turbulence [4]. The skin of fast-swimming sharks, characterized by a microstructure of riblets aligned with the direction of flow, has also shown a capability to reduce skin friction drag in fully turbulent flow.

Another field of study within biomimetics is focused on understanding the mechanisms leading to fouling resistance on the natural surfaces of several plants and animals. Surface fouling is the unwanted accumulation of environmental matter on a surface, leading to a loss of its intended function. In general terms, fouling is used to describe both the accumulation of organic matter and inorganic matter. For instance, the lotus leaf exhibits a ‘self-cleaning’ capability whereby previously accumulated dirt and dust particles are carried away with droplets of rainwater rolling off its surface. The mechanism responsible for this behavior is the microstructure covering the surface of the leaf, ultimately leading to its superhydrophobicity. An alternative to self-cleaning is to prevent the initial accumulation altogether. This is known as ‘fouling resistance’ and is demonstrated by many insects, birds, various species of mollusks, fish, and marine mammals. Although nature employs a diverse means of achieving fouling resistance, the observed mechanisms may be categorized as chemical, physical, or mechanical [5]. These mechanisms may be employed individually or in conjunction with one another to achieve a defense solution tailored to specific environments.

While nature employs tailored solutions to deal with environmental foulants, surface fouling remains a persistent challenge for many applications, including marine transportation, medical systems, and industrial operations like pipelines and water purification [6,7,8,9,10]. Ship hull fouling is estimated to result in an annual global cost in excess of $150 billion USD due to increased fuel consumption, cleaning, and maintenance activities [6,11]. The fouling process starts with the formation of a biofilm composed of various proteins and macromolecules. This initial layer creates an opportunity for more complex fouling organisms—such as barnacles and mussels—to attach and grow [12]. The social cost of ship hull fouling must also be considered, since much of the macrofouling on ship hulls consists of live organisms that may become invasive species in destination waterways [13]. Environmentally friendly paints and coatings are currently in use to combat fouling but they all have limitations [14]. As such, ship hulls are periodically cleaned with pressure washers and mechanical scrubbing followed by reapplication of a fouling inhibitor. Additionally, bacterial colonization on medical devices such as implants and surgical equipment, represents an important area of research due to its direct impact on human well-being. It is estimated that medical devices annually cause more than 2 million infections, and 5000 deaths [15].

Nonetheless, the mechanisms found in nature could offer efficient ways to attain resistance against fouling for the industries that are still grappling with surface fouling. Along these lines, the shells of 36 species of mollusks were investigated for their fouling resistance and self-cleaning capabilities relative to a 60 mm × 30 mm reference sample of polyvinylchloride (PVC) [16]. The surface characteristics of each specimen were also investigated using laser scanning confocal microscopy (LSCM) along with scanning electron microscope (SEM) imaging. Samples were submerged for a total of 12 weeks in the outflow of Ross Creek in Townsville, Australia. Surface fouling was quantified on a bi-weekly basis with an image analysis program and defined as the percentage of the surface covered with environmental foulants. The most fouling-resistant mollusk species identified by this study was the bivalve *Dosinia juvenilis* (DJ), showing 29.0% fouling coverage after the 12-week trial.

Each specimen was then subjected to a jet of water according to a technique developed to approximate the wall shear stress on ship hulls travelling at 25–35.5 knots. This allowed for quantification of the self-cleaning capabilities of each specimen. The bivalve, *Septifer bilocularis* showed a reduction of 99.3% in fouling coverage, the highest performing species. It was found that 86.3% of the fouling was removed from *Dosinia juvenilis*, which gave it a noteworthy ranking of sixth in the self-cleaning study. Of note, these organisms lack chemical and physical mechanisms for achieving fouling resistance and self-cleaning. Instead, their surfaces exhibit intricate topographies featuring micro- and nano-scale geometrical features. These features, which are visually unique to each species, serve as natural and passive methods for fouling resistance and self-cleaning. Such passive means of achieving fouling resistance remain attractive for large-scale engineering applications such as the challenge for ship hulls previously mentioned.

To understand the functional mechanisms leading to the observed fouling resistance of the outer surface of the *Dosinia juvenilis*, the present study aimed to investigate the hydrodynamics of the surface topography. The fouling-resistant performance of shark skin has been attributed to the mechanisms resulting in its capacity to reduce skin friction drag. More specifically, it is believed that the hydrodynamic effects of drag reduction allows for a rapidly moving water layer located near the structured surface that carries away particles that would otherwise settle and adhere to the surface [17,18]. Furthermore, if nature employs a fundamental relationship between fouling resistance and drag reduction, it can be anticipated that the fouling-resistant topography of the *Dosinia juvenilis* also possesses an ability to reduce the drag associated with the turbulent coastal waters of the species’ natural environment [16]. 

Therefore, gaining a more comprehensive understanding of the hydrodynamics in turbulent flow near the surface of *Dosinia juvenilis* is anticipated to offer insights into the origins of its fouling resistance capabilities. To address this, the main goal of the present study was to conduct a numerical examination of the drag reduction mechanisms associated with bio-inspired riblets. These riblets were based on surface scans of biological samples from *Dosinia juvenilis*. This study aimed to compare the flow characteristics of these riblets with those of similarly sized shark-inspired riblets, which have been extensively researched in the past [19].

## 2. Analysis of the Geometrical Features of *Dosinia juvenilis*

**Figure 1:**
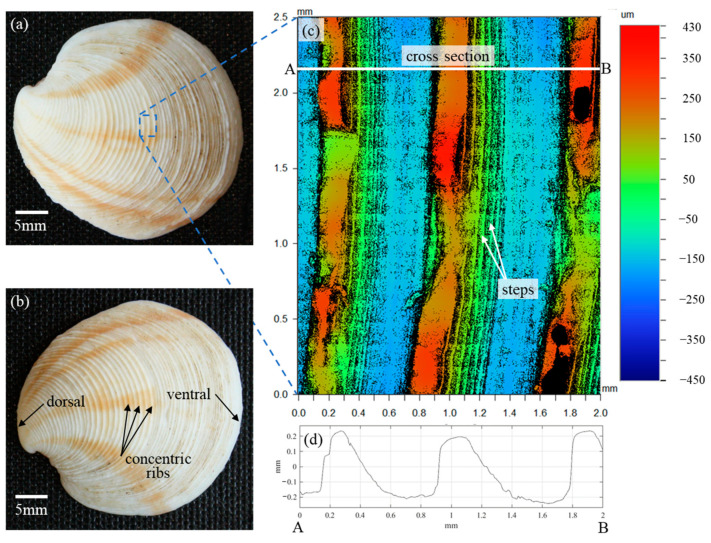
Details of the mollusk *Dosinia juvenilis*: (**a**,**b**) images outlining pertinent anatomical features, (**c**) areal surface topography, and (**d**) profile view along line AB.

In the analysis of Scardino et al. [16], the fouling resistance performance of the 36 mollusk species was solely attributed to the distinct characteristics of the surface textures. Reverse engineering the species with the highest fouling resistance performance, *Dosinia juvenilis*, was expected to result in a better understanding of the fundamental mechanisms of fouling resistance as it relates to surface topography. Reverse engineering is only enabled by first understanding the surface characteristics particular to this species. While the previous study made use of laser scanning confocal microscopy (LSCM) to acquire three-dimensional (3D) surface scans, the scanned area of the shell surface was just 79 µm × 79 µm. In contrast to the approximately 800 µm periodicity of the *Dosinia juvenilis* ribs, the scanned area was not sufficient to reverse engineer the surface for the purpose of the present study. As such, three specimens of *Dosinia juvenilis* were utilized to provide a precise understanding of the surface characteristics of this species. Optical images of the specimens were acquired with a CCD camera (Figure 1a,b), and surface topographies were captured using a Wyko optical profilometer. The surface topography of the specimens was scanned with a lateral resolution of 384 nm along the X- and Y-axes (i.e., a plane parallel to the surface), and with a vertical resolution of 0.1 nm along the Z-axis defined as being perpendicular to the surface). The image displayed in Figure 1c represents a 2.0 mm × 2.5 mm composite of 170 individual 246 µm × 184 µm sub-area scans, stitched together to form a single contour plot, with colors representing the topography height.

According to the bivalve morphology, the outer shell surface is referred to as the periostracum and is composed of an organic matrix consisting of calcium carbonate crystals bound together with conchiolin, a complex protein [20]. The dorsal side of the shell is located near the hinge, with the ventral side being opposite to this, and typically where the shell opens. The findings of Scardino et al. demonstrate that the surface topography on the periostracum shows incredible variety between species, ranging from smooth surfaces to ordered textures described as spines, perforations, and ribs similar to those depicted in Figure 1 [16]. Scardino et al. also demonstrated that the microtopography and composition of the surface greatly influence the contact angle of water droplets, with a range of 18° to 81°. However, the contact angles did not contribute to fouling resistance or self-cleaning in a statistically significant manner. The correlation coefficient between values of the contact angles and fouling resistance coefficients was ${\left(r_{xy}\right)}_{fr}=-0.18$ and ${\left(r_{xy}\right)}_{sc}=0.08$ for self-cleaning, where the subscripts *fr* and *sc* denote fouling resistance and self-cleaning, respectively. These findings suggest the observed fouling performance may be attributed to the surface micro/nano-topography alone.

The largest structures on the periostracum of the *Dosinia juvenilis* are visible to the naked eye and characterized as concentric ribs emanating from the dorsal end (i.e., near the hinge). The crest of each rib demonstrates a seemingly random variation in height along its length with little correlation to the adjacent crests. Whether this variation was present at the formation of this specimen or was a result of wear over the lifetime of the creature is unclear. The surface is also hierarchical in nature with structures on the sloping faces of each rib composed of several “steps” at approximately 50 µm intervals. The rib cross-section reveals an asymmetric shape with a steep inclination angle on the dorsal side of the crest, and a more moderate angle on the ventral side of the crest Figure 1d. Spacing between each rib is approximately 800 µm with plateaus in the valley of greater length than at the crest.

**Figure 2:**
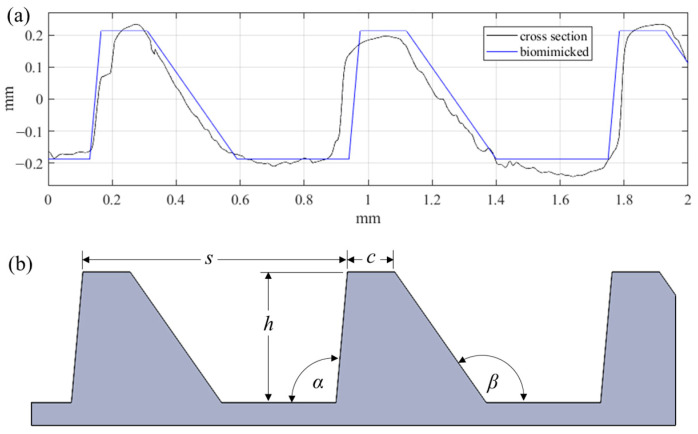
(**a**) Comparison of biomimicked geometry with DJ cross section, and (**b**) parameters defining the biomimicked geometry.

Similarly to what is seen throughout nature, this surface exhibits a high degree of irregularity (i.e., randomness) within the periodic structures. Due to the hierarchical and stochastic nature of this geometry, replicating the surface for large scale applications with modern manufacturing tools represents a significant challenge. The surface hierarchy and irregularities are difficult to reproduce. Instead, individual surface characteristics should be considered alone as research efforts continue toward understanding how best to achieve fouling resistance, and to provide a means of forecasting the performance of a given surface. For these reasons, the proposed riblet geometry was bio-inspired by the ribs of the *Dosinia juvenilis* as it is presented in Figure 2.

The asymmetric ribs of the bio-inspired geometry have a spacing (*s*) of 810 µm, and height (*h*) of 400 µm. The angle on the dorsal facet (*α*) is 95°, while the angle of the ventral facet (*β*) is 125°. Finally, the width of the crest (*c*) was defined as 145 µm. These five parameters fully define the form geometry. Unlike the natural surface, the cross-section irregularities and rib curvature were neglected in this preliminary study. This novel surface form topography was designed with the intention of using micromilling processes for proven surface texturing, structuring, and microfabrication [21,22].

Riblet geometries have been studied extensively in the past for their ability to reduce drag in a fully turbulent flow with mean velocity parallel with the riblet crests and valleys [23]. Riblets with characteristic dimensions tailored to the flow conditions have a demonstrated ability to reduce the velocity fluctuations near the surface, within the valley between ribs. The result is a surface that exhibits a reduction in fluid drag relative to a flat reference surface despite a net increase in the wetted surface area. The proposed riblet geometry shares several characteristics with the shark-inspired riblets such that in seeking to elucidate the functional mechanisms employed by DJ toward fouling resistance, a relative comparison was made with 60° sawtooth riblets in the present study. Similarly to other riblet surfaces, the sawtooth riblets have demonstrated, experimentally and numerically, a maximum drag reduction potential of 6% [23]. The use of riblets by sharks as well as DJ and other bivalve species give further merit to the hypothesis that nature makes use of such structured surfaces not just to manipulate the drag, but also to modify its fouling characteristics. As research on naturally occurring riblet surfaces continues, it is expected that further functionality provided by such structures will be revealed.

While bivalves do not rely on swimming like other fish, they may still benefit from a reduction in drag to help them stay in one spot in coastal waters. Also, the flow characteristics over the surface may provide a washing effect to help the bivalve remain clean.

## 3. Numerical Modeling

This section will outline the comparison modeling of the proposed, novel geometry with the sawtooth riblet geometry at identical periodicity, or spacing between riblets (*s*). The sawtooth geometry was investigated at two riblet spacings corresponding to known regions leading to (1) a reduction in drag and (2) an increase in drag. The DJ geometry was also investigated at these two spacings to allow for a direct comparison of the flow statistics and drag reduction performance. Due to the novel nature of the proposed DJ geometry, an additional six numerical simulations were carried out, providing data for a drag reduction versus spacing curve. It is expected that through investigation of the turbulence statistics near the surface of the proposed geometry, further insight of its ability to deter surface fouling will be gained. 

### 3.1. Methodology and Applied Techniques for Numerical Simulation Methods

Numerical simulations were run on a compute cluster with 128 CPU cores. Each transient simulation took approximately 48 h to complete. Flow over the riblets was simulated using ANSYS Fluent along with the large eddy simulation (LES) model. This model has been used in the past, displaying excellent correlation with the experimental data [24,25].

LES directly resolves large-scale turbulence while applying a model to the smallest scales to reduce computational efforts. The smallest scales of the chaotic turbulent beha-vior contain the least amount of energy yet are most computationally difficult to resolve because of the corresponding small size of the computational cells required. Also, at the smallest scales, turbulent eddies are largely isotropic and do not rely on local flow conditions [26]. As such, a model can be applied to the sub-grid scales (i.e., sub-grid scale model). The Wall-Adapting Large Eddy (WALE) model was used in this case.

### 3.2. Computational Domain and Spatial Discetization

**Figure 3:**
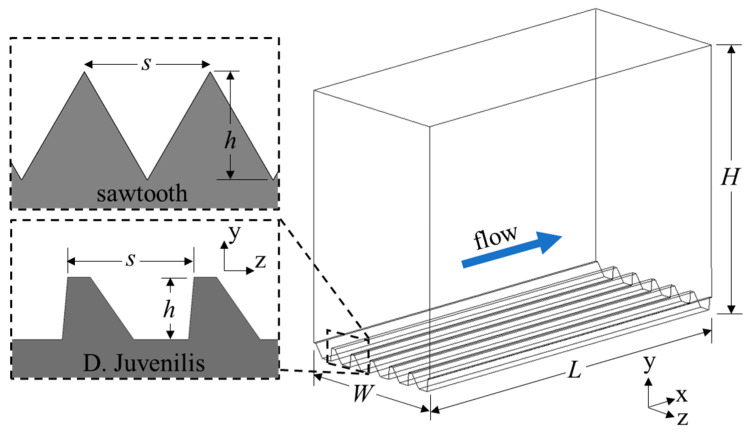
Computational domain and coordinate system for the drag-increasing case of the DJ and sawtooth geometry.

The computational domains were created with the riblet surfaces at the bottom and a flat surface on the top wall to serve as a reference for calculating drag reduction (Figure 3). These two surfaces had a no-slip boundary condition assigned to them, while a periodic condition was applied to the inlet and outlet as well as the left and right surfaces. A periodic boundary allows fluid to pass through the surface, being introduced on the opposite face. In this way, the domain behaves as a semi-infinite domain allowing turbulence to propagate throughout the domain, interacting only with the top and bottom surfaces. This specific arrangement is known as plane channel flow and has been used extensively in similar studies. The length (*L*), height (*H*), and width (*W*) of the computational domain were chosen to be larger than the expected scales of the coherent flow structures and to adhere to the minimal flow unit described by Jimenez et al. [27] to ensure a fully turbulent flow field was accurately modeled.

Simulation results have shown that maximum drag reduction for sawtooth riblets is achieved at a non-dimensional spacing (*s^+^*) of approximately 16 according to:

(1) $$s^{+}=s\cdot \frac{u_{\tau }}{\nu }$$

Here, *s* represents the dimensional spacing, *ν* is the fluid kinematic viscosity, and $u_{\tau }$ is the friction velocity determined as:

(2) $$u_{\tau }=\sqrt{\frac{\tau_{0}}{\rho }}$$

Here, *ρ* is fluid density and $\tau_{0}$ *is* shear stress on the flat reference surface. 

It is worth noting that *s^+^* non-dimensionalization enables the calculation of appropriate physical dimensions of the riblet geometry based on the flow conditions near the structured surface. Identical flow conditions were used throughout the numerical simulations investigated, representing a fully turbulent flow of water in a channel at *Re* = 4200, resulting in a wall shear stress $\tau_{0}$ of approximately 1.25 Pa. Flow field comparisons between the two geometries at dimensionless spacings of *s^+^* = 16 (for drag-decreasing) and *s^+^* = 36 (for drag-increasing) cases, corresponding to spacings of 462 µm and 1000 µm, respectively. While this spacing represents a departure from what was measured on biological samples of DJ, a direct comparison to the flow over sawtooth riblets of similar dimensions helps reveal why nature employs the asymmetric riblets seen on the biological specimen.

The computational domains were each discretized with approximately 3.0 × 10^6^ hexahedral elements biased toward the no-slip boundaries in order to conform to the best practices [26]. More specifically, for the boundary layer to be accurately resolved, the first cell at a no-slip boundary must be adhere to *y^+^* ≤ 1, determined as:

(3) $$y^{+}=y\cdot \frac{u_{\tau }}{\nu }$$

A Reynolds number (*Re*) of 4200 was achieved by applying a constant streamwise (*x*-direction) flow to the domain according to:

(4) $$Re=U_{l}\frac{\delta }{\nu }$$

where *U_l_* is the centerline velocity of a parabolic laminar profile with the same volume flowrate, *Q*, and *δ* is the channel half-height (*H*/2). In the present work, *U_l_* was targeted at approximately 0.84 m/s. Finally, the volume flow rate can be calculated with:

(5) $$Q=\frac{2}{3}AU_{l}$$

Here, *A* represents the cross-sectional area of the channel in the *yz-*plane. The simulations were initialized according to best-practices by solving a steady-state simulation, using a time-averaged model, followed by superimposing turbulent fluctuations. The steady-state solution then served as the initial conditions of the transient simulation. The simulation was advanced in time to allow for natural turbulence to develop and reach a steady state while monitoring wall shear stress at the top and bottom walls. The runs had a computational time step of 8 × 10^−5^ s in keeping with a Courant number of one. Each simulation required approximately 3.0 s of simulation time to reach steady conditions. Following this, the simulation was then further advanced in time to allow for time averaging of statistical quantities. In a similar fashion to the work of Choi et al. [28], at least 500 non-dimensional time steps (*T^+^*) were used for time averaging statistical quantities where:

(6) $$T^{+}=T\frac{U_{l}}{\delta }$$

These conditions resulted in time averaging over more than 3.0 s or 37,500 time steps.

### 3.3. Initial Validation: Sawtooth Riblets Model

In order to validate the applicability of LES coupled with the WALE sub-grid scale model to turbulent flow over riblets, two numerical simulations were performed on 60° sawtooth riblets with *s^+^* of 16 and 36, identical to that used for the proposed DJ geometry. In closed channel experiments, Bechert et al. [23] investigated the drag reduction performance of 60° sawtooth riblets for 6 ≤ *s^+^* ≤ 38. A drag reduction of 4.8% was demonstrated at *s^+^* 16, while a drag increase of 11.0% was achieved at *s^+^* 36. Choi et al. [28] performed a direct numerical simulation of 60° sawtooth riblets at *s^+^* 20 and 40, similarly corresponding to drag-decreasing and drag-increasing cases, respectively. The results from previous studies on sawtooth riblets will be compared with those of this current study. This comparison aims to validate the findings and consequently, enhance the confidence in the outcomes related to the proposed DJ geometry and the derived conclusions.

## 4. Results and Discussion

In this section, the simulation results of the *Dosinia juvenilis-*inspired surface will be investigated and compared to the sawtooth riblet results. The change in drag relative to a flat surface will first be presented, followed by a statistical analysis of the respective flow fields to decipher the mechanisms leading to the drag reduction performance.

### 4.1. Change in the Drag Performance

The instantaneous friction drag over a generic surface was calculated as the integral of the differential wall shear stress over the entire wetted surface [29] as follows:

(7) D=∫τdA

The instantaneous change in drag with respect to a flat surface [27] is given by:

(8) $$\Delta D=\frac{D_{r}-D_{f}}{D_{f}}$$

where *r* and *f* denote riblet and flat surfaces, respectively. According to Equation (7), a drag-decreasing surface will report a negative value for Δ*D.*

**Figure 4:**
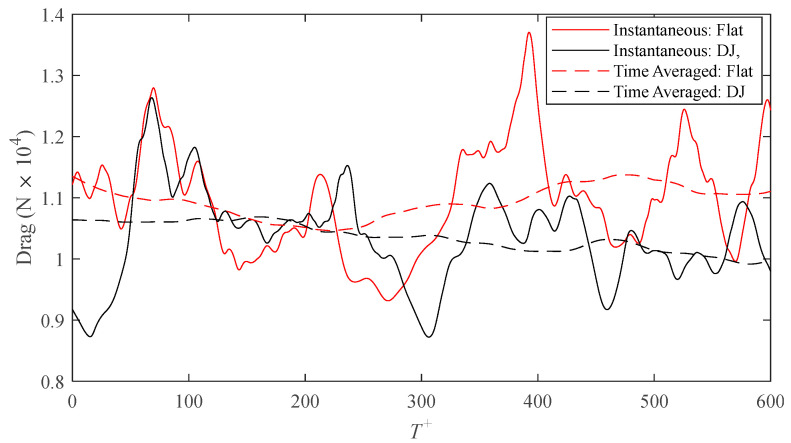
Time history of the drag force on the riblet and smooth surfaces for the *s*^+^ = 16 case of *D. juvenilis*.

Due to the difference in wetted surface area between the flat and ribletted surfaces, direct comparisons of drag forces on each surface are more appropriate than comparisons of shear stress alone (because they both share the same projected area). Figure 4 shows the intermittent behavior of the drag on each surface for the drag-decreasing case, *s^+^* = 16, of the DJ geometry. The area integral used to calculate the drag force is analogous to an areal average of the drag. As such, a larger surface area (i.e., larger domain) would dampen the intermittency of the fluctuations. However, enlarging the domain would require more computational cells for discretization, which would in turn increase the computational workload. Instead, the performance of the surface is more effectively illustrated by application of a centered moving average with a window size of *T^+^* = 250. Except for a brief period of time, the averaged drag on the DJ surface is consistently lower than that on the flat surface. A time average over the complete series (*T^+^* = 600) results in a drag reduction of 6.1% calculated by means of Equation (7) where the time averaged drag on each surface is calculated as follows:

(9) $$\bar{D_{r}}=\frac{1}{T}\sum_{t=0}^{T}D_{r}$$

**Figure 5:**
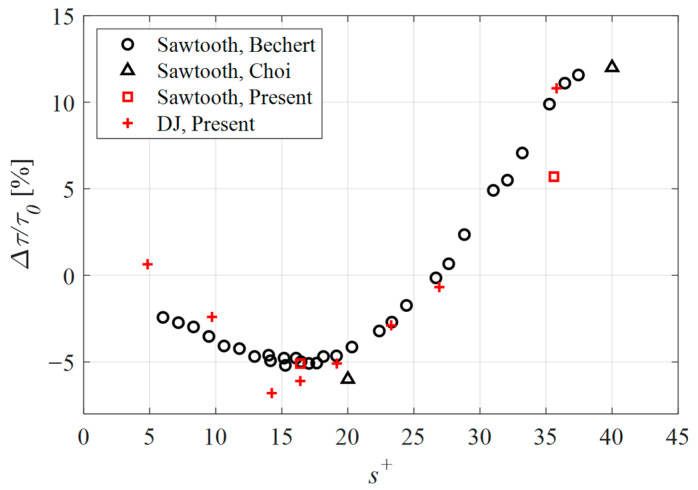
Drag change for the sawtooth and DJ cases considered in the present study compared with the numerical data of Choi et al. [28] and empirical results obtained by Bechert et al. [23].

In Figure 5, the drag change for the cases considered in the present study is presented together with previously published data. According to this plot, the sawtooth geometry demonstrated a drag reduction of 5.1% and a drag increase of 5.7% for the *s^+^* = 16 and *s^+^* = 36 cases, respectively. While the drag reduction case agrees well with the published data, the expected performance for the *s^+^* = 36 case was a drag increase of approximately 11%. The difference in performance between the present numerical simulations and the empirical work by Bechert et al. [23], was attributed to the surface roughness resulting from the fabrication process. More specifically, in case of numerical simulations, the walls were modeled as being perfectly smooth and therefore without any surface roughness. The drag caused by a surface roughness increases with turbulence. As such, the taller riblets of the drag-increasing case (*s^+^* = 36) penetrate further into the turbulent flow where the surface roughness of the fabricated samples acts to increase drag more so than was achieved numerically. By contrast, the shorter, drag-reducing riblets do not penetrate into the turbulent flow to the same extent; therefore, the impact of surface roughness was not as pronounced.

The DJ geometry outperformed the sawtooth riblets with an achieved drag reduction of 6.1% for the *s^+^* = 16 case, and a maximum drag reduction of 6.8% for the smaller spacing of the *s^+^* = 14 case. At the largest spacing considered (*s^+^* = 36), the drag relative to a flat surface was 10.8%. The remaining data points demonstrate that the proposed geometry performs in a similar manner to the sawtooth riblets, and by extension, similar to riblet geometries in general.

### 4.2. Analysis of Turbulent Statistics

For comparison purposes, the turbulent statistics were obtained for four of the present cases associated with *s^+^* = 16 and *s^+^* = 36 for both the sawtooth and DJ geometry. The turbulent statistics were quantified over the same time interval used for determining friction drag—at least *T^+^* = 500. The velocity at each cell is a vector summation of three components, *U*, *V*, and *W*, corresponding to the three cartesian coordinates, *x*, *y*, and *z*, respectively. Each component is comprised of the mean velocity ($\bar{U}$) and the fluctuating component (*u*′) according to the following relationship:

(10) $$U=\bar{U}+u^{\prime }$$

The fluctuating component is a property of the turbulent flow, such that without fluctuations, the flow is considered laminar. Velocity fluctuations are also the cause of the increased shear stress on a surface (i.e., friction drag, or skin friction) compared to a laminar flow at the same velocity. Several studies have demonstrated that the ability of shark-inspired riblets to reduce friction drag is a direct result of decreased velocity fluctuations near the surface [30]. More specifically, riblets have been shown to decrease the wall-normal and spanwise velocity fluctuations perpendicular to the mean flow, $v^{\prime }$ and $w^{\prime }$ in this case. The square root of variance (i.e., root mean square) is typically used to describe the intensity of velocity fluctuations about the mean in each direction and is denoted by the subscript *rms*. In the case of the streamwise velocity fluctuations, *u_rms_* (velocity fluctuations in the *X*-direction), the equation for a discrete set of *N* points is given by:

(11) $$u_{rms}=\sqrt{\frac{1}{N}\sum_{i=1}^{N}{u_{i}^{\prime }}^{2}}$$

**Figure 6:**
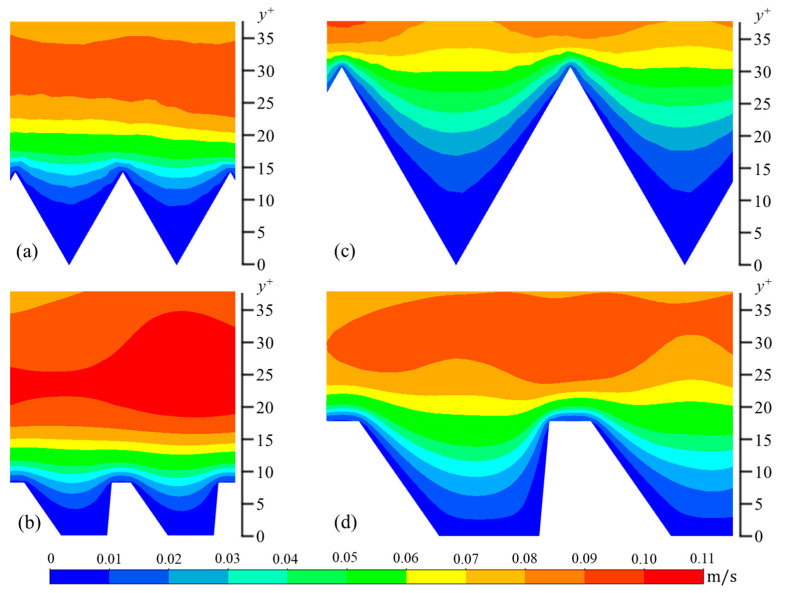
Contour plots of streamwise velocity fluctuations (*u_rms_*): (**a**) *s^+^* = 16 sawtooth, (**b**) *s^+^* = 16 DJ, (**c**) *s^+^* = 36 sawtooth, and (**d**) *s^+^* = 36 DJ.

**Figure 7:**
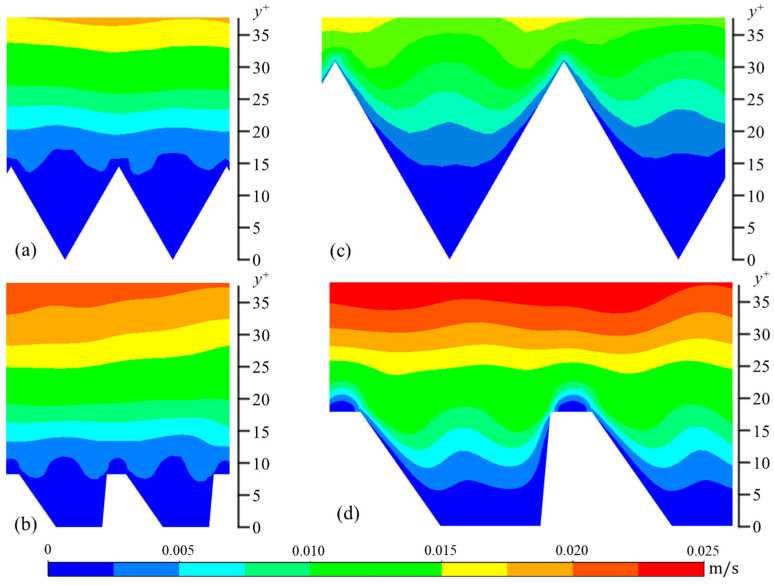
Contour plots of wall-normal velocity fluctuations (*v_rms_*): (**a**) *s^+^* = 16 sawtooth, (**b**) *s^+^* = 16 DJ, (**c**) *s^+^* = 36 sawtooth, and (**d**) *s^+^* = 36 DJ.

**Figure 8:**
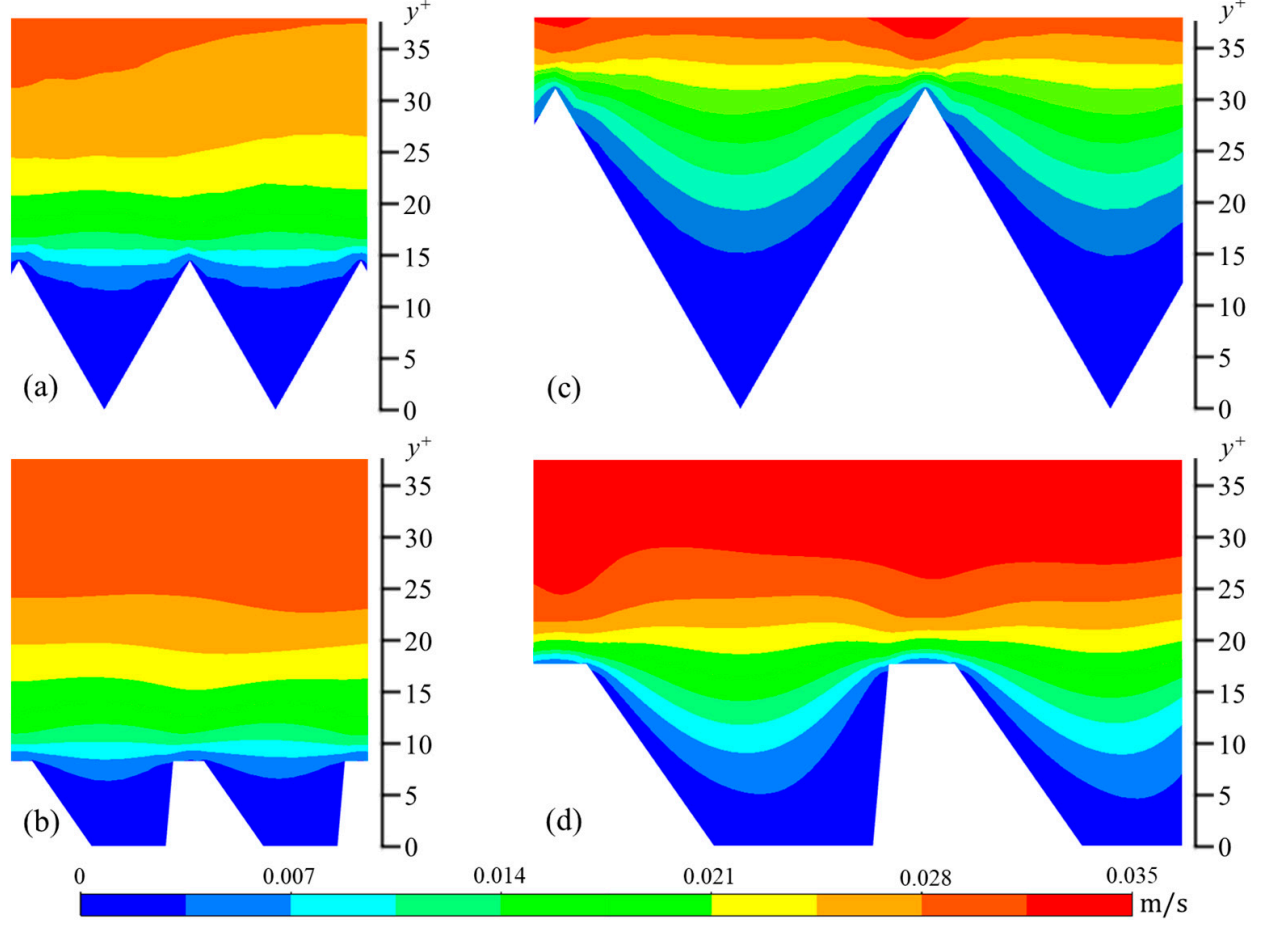
Contour plots of spanwise velocity fluctuations (*w_rms_*): (**a**) *s^+^* = 16 sawtooth, (**b**) *s^+^* = 16 DJ, (**c**) *s^+^* = 36 sawtooth, and (**d**) *s^+^* = 36 DJ.

The wall-normal (*v_rms_*) and spanwise (*w_rms_*) components are calculated in a similar manner. In Figure 6, Figure 7 and Figure 8, contour plots of velocity fluctuations have been shown on a y-z plane perpendicular to the flow direction. For the drag-reducing cases of both geometries, the velocity fluctuations within the riblet valleys are relatively small compared to those of the drag-increasing cases (Figure 6, Figure 7 and Figure 8). Velocity fluctuations in both geometries penetrate equally as far into the riblet valleys, providing evidence for their similar drag-reducing performance. For the drag-increasing case of DJ, all three components of the velocity fluctuations penetrate deeper into the riblet valley than they do for the drag-increasing sawtooth case. This observation coincides with the increased drag of DJ compared to sawtooth (10.8% compared to 5.7%). It is evident here that the lower *h*/*s* ratio of DJ, along with its flat valleys, enables the turbulent fluctuations to penetrate more freely into the riblet valleys.

The contour plots of the wall-normal (*y*-direction) velocity fluctuations show a unique behavior in all four cases (Figure 7). More specifically, in the center of each riblet, the contour lines have a convex shape, suggesting the vertical velocity fluctuations are weaker here than near the inclined facets of the riblets. This convex shape supports the observation that drag-reducing riblets decrease turbulent fluctuations near the surface by preventing streamwise vortices from descending into the riblet valleys. This has been demonstrated both numerically and empirically [30]. Streamwise vortices are described as intermittent regions of fluid rotating around an axis parallel to the streamwise direction. As such, they have velocity components *V* and *W*. The characteristic diameter of the near-wall vortex is approximately 30 non-dimensional wall units with its center located on average at *y^+^* = 20 [24,28,31]. Therefore, at riblet spacings less than *s^+^* = 30, the vortex is unable to descend into the riblets, while for spacings larger than this, vortices penetrate into riblets, and, as a result, increase friction drag.

**Figure 9:**
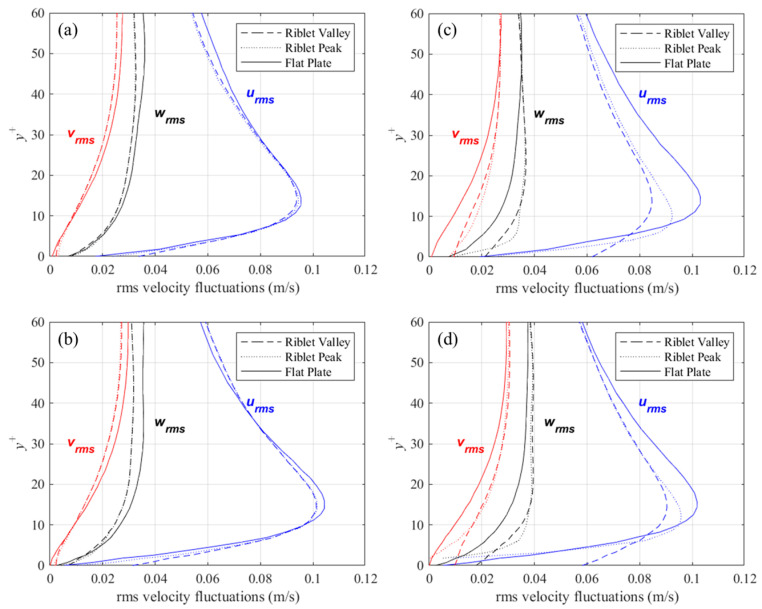
Mean rms velocity fluctuations above riblet tips and valleys compared to a flat surface for: (**a**) *s^+^* = 16, 60° sawtooth, (**b**) *s^+^* = 16, *D. juvenilis*, (**c**) *s^+^* = 36, 60° sawtooth, and (**d**) *s^+^* = 36, *D. juvenilis*.

Profiles of root-mean-square (*rms*) velocity fluctuations above the riblet tips, valleys, and above a flat surface are shown in Figure 9. The data for these plots are averaged for each riblet and in *x*, and thus better represent a the mean rms velocity fluctuations at the corresponding locations. Also, to compare flow above riblets with a flat surface, the concept of a virtual origin must be introduced. The virtual origin for the riblets has been defined several ways in the past. In this study, the definition proposed by Choi et al. [28] was adopted. The virtual origin is as an *xz*-plane placed at a *y^+^* location where an imaginary flat surface would produce the maximum streamwise velocity fluctuations at the same *y^+^* location. The location of the virtual origin for each of the four cases considered is presented in Table 1, along with the height and spacing in non-dimensional wall coordinates.

For the drag-reducing cases (i.e., *s^+^* = 16) of the two riblet configurations, velocity fluctuations perpendicular to the streamwise direction are diminished relative to the flat surface, while the streamwise fluctuations show little change. For the larger, drag-increasing riblets, there is a significant increase in perpendicular velocity fluctuations within the riblet valleys. By contrast, the streamwise velocity fluctuations show a marked decrease. This behavior is slightly diminished for the deeper sawtooth riblets than for DJ, thus suggesting that shallow riblets facilitate the penetration of velocity fluctuations. A similar behavior was reported by Choi et al. [28] for riblets with *s^+^* = 40: a large decrease in streamwise velocity fluctuations above the riblet valleys was not noticed for the drag-decreasing case.

### 4.3. Mechanisms of Drag Reduction and Fouling Resistance

Drag reduction for the riblets aligned with the streamwise flow direction is the result of suppression of near-surface velocity fluctuations and prevention of the near-wall streamwise vortex from penetrating into the grooves. Previous studies have shown that drag reduction is achieved for spacings up to *s^+^* ≈ 25; however, the maximum drag reduction effect is achieved at approximately *s^+^* ≈ 16 depending on the specific riblet geometry. Above *s^+^* ≈ 25, the drag reduction effect is diminished and the friction drag at the surface begins to behave according to typical surface roughness theory [32].

The height to spacing ratio, *h*/*s*, of 60° sawtooth riblets is approximately 0.87 whereas the bio-inspired DJ riblets is 0.49. Therefore, at a given spacing, the sawtooth riblets extend further into the boundary layer flow, exposing the tips to higher velocities and associated shear stress. This offers a potential explanation for the small drag reduction performance advantage the DJ riblets have over the sawtooth riblets. Furthermore, the wetted surface area of the DJ riblets is 14.5% less than the 60° sawtooth riblets. When exposed to the same shear stress, it is advantageous to have less surface area for the shear stress to act on. Furthermore, the flat cap width, *c,* of the bio-inspired DJ riblets also provides additional strength and durability over the tips of the sawtooth riblets. This characteristic makes the geometry more appealing for harsh environments where the effects of impact and abrasion may decrease the surface longevity.

The presence of drag reduction and fouling resistance on some species of sharks has provided for the hypothesis of this research: an intrinsic correlation exists between drag reduction and fouling resistance that is employed throughout the natural world. It may be argued that drag-reducing surfaces result in less turbulent activity near the surface, thus reducing fouling because of the impaction of particles suspended in the flow with the surface. An opposing argument suggests that a drag-increasing surface may lead to fouling resistance because the increased shear stress may overcome adhesion forces.

Alternatively, fouling resistance for some species may not depend on the fluid dynamics over the structured or textured surfaces. Instead, the characteristic dimensions of the natural riblet surface may be “tuned” to the dimensions of the typical fouling media found in the environment. This relationship has been investigated for organic fouling by aquatic organisms. For instance, several studies on the settlement of zoospores of green *alga ulva* on various topographies concluded that characteristic dimensions play a critical role in the settlement of such small, motile organisms. The optimal structure sizes for reducing settlement of this particular organism were on the order of 2 µm, approximately the same diameter of the organism [33,34,35]. With valley widths approximately 600 µm, the riblets of the *D. juvenilis* are approximately 300 times larger than the 2 µm zoospores, so they would not prove effective according to the findings of those studies.

## 5. Summary, Conclusions, and Future Work

The unique structures on the periostracum of the bivalve *Dosinia juvenilis* have been shown in the past to exhibit superior fouling resistance and self-cleaning capabilities. To investigate the mechanisms leading to such performance, turbulent flow was numerically simulated over a novel riblet geometry inspired by *Dosinia juvenilis* and compared to 60° sawtooth riblet geometry displaying similar dimensions and characteristics. Large eddy simulations of turbulent flow over the riblets were carried out at a Reynolds number of 4200. The drag was recorded at each time step of the transient simulation in order to calculate the change in drag relative to the flat reference surface. A maximum drag reduction of 6.8% was displayed for the s^+^ = 14 case of the bio-inspired geometry. Flow statistics leading to drag reduction were similar to those of the sawtooth riblets and, by extension, other riblet geometries in parallel flow. More specifically, riblets have a constraining effect on the near-surface turbulent eddies, lifting them away from the surface, thereby reducing the friction drag associated with turbulence. As a result, the transverse and wall-normal velocity fluctuations above the riblet surface were reduced in the drag-reducing configuration.

The result of this work suggests that nature indeed makes use of riblet surfaces with dimensions tailored to the environmental conditions toward multiple functionalities. In the past, the biological surface of *Dosinia juvenilis* has demonstrated a superior ability to reduce fouling, and it has been shown in the present work that the riblets bio-inspired by its surface may be able to reduce drag. A parametric investigation of the five characteristic dimensions of the geometry (*s*, *h*, *α*, *β*, and *c*) may reveal the optimal shape of the trapezoidal groove for drag reduction performance.

Since the proposed DJ riblet geometry has demonstrated a drag reduction potential, future studies will focus on experimentally investigating the fouling resistance potential to (1) examine the correlation between drag reduction and fouling resistance, and (2) determine if the bio-inspired geometry possesses the same ability to resist fouling as its biological counterpart. Such investigations will require precision fabrication and the development of a strategy to accurately replicate the bio-inspired geometry. Future work will also focus on the development of an experimental apparatus that enables fouling experiments to be conducted at flow conditions known to be within the drag reduction regime of the geometry. In this way the anticipated correlation between drag reduction and fouling resistance can be investigated explicitly.

## Figures and Tables

**Table 1:** Location of the virtual origin for each riblet case.

Case	Spacing (*s*^+^)	Height (*y*^+^)	Virtual Origin (*y*^+^)
Sawtooth, drag-reducing	16.4	14.2	15.2
DJ, drag-reducing	16.6	8.2	8.6
Sawtooth, drag-increasing	35.6	30.8	30.6
DJ, drag-increasing	35.7	17.6	16.4

## Data Availability

Data are contained within the article.
